# Advancement of non-destructive spectral measurements for the quality of major tropical fruits and vegetables: a review

**DOI:** 10.3389/fpls.2023.1240361

**Published:** 2023-08-16

**Authors:** Umuhoza Aline, Tanima Bhattacharya, Mohammad Akbar Faqeerzada, Moon S. Kim, Insuck Baek, Byoung-Kwan Cho

**Affiliations:** ^1^ Department of Agricultural Machinery Engineering, Chungnam National University, Daejeon, Republic of Korea; ^2^ Department of Smart Agricultural Systems, Chungnam National University, Daejeon, Republic of Korea; ^3^ Environmental Microbial and Food Safety Laboratory, Agricultural Research Service, United States Department of Agriculture, Beltsville, MD, United States

**Keywords:** non-destructive measurement, spectral measurements, quality parameters, tropical fruits and vegetables, rapid measurement

## Abstract

The quality of tropical fruits and vegetables and the expanding global interest in eating healthy foods have resulted in the continual development of reliable, quick, and cost-effective quality assurance methods. The present review discusses the advancement of non-destructive spectral measurements for evaluating the quality of major tropical fruits and vegetables. Fourier transform infrared (FTIR), Near-infrared (NIR), Raman spectroscopy, and hyperspectral imaging (HSI) were used to monitor the external and internal parameters of papaya, pineapple, avocado, mango, and banana. The ability of HSI to detect both spectral and spatial dimensions proved its efficiency in measuring external qualities such as grading 516 bananas, and defects in 10 mangoes and 10 avocados with 98.45%, 97.95%, and 99.9%, respectively. All of the techniques effectively assessed internal characteristics such as total soluble solids (TSS), soluble solid content (SSC), and moisture content (MC), with the exception of NIR, which was found to have limited penetration depth for fruits and vegetables with thick rinds or skins, including avocado, pineapple, and banana. The appropriate selection of NIR optical geometry and wavelength range can help to improve the prediction accuracy of these crops. The advancement of spectral measurements combined with machine learning and deep learning technologies have increased the efficiency of estimating the six maturity stages of papaya fruit, from the unripe to the overripe stages, with F1 scores of up to 0.90 by feature concatenation of data developed by HSI and visible light. The presented findings in the technological advancements of non-destructive spectral measurements offer promising quality assurance for tropical fruits and vegetables.

## 1 Introduction

Tropical fruits and vegetables are agricultural crops that are typically grown in tropical regions where the climate is warm, with temperatures ranging from 20 to 35^0^C (Bahadur et al., 2020). Tropical regions are found amidst the tropics of Cancer and Capricorn, and encompass equatorial zones in Oceania, Asia, Africa, Central and South America, and the Caribbean (Zakaria, 2023). Crops grown naturally in such weather conditions provide essential minerals, water, fiber, and vitamins that contribute significantly to the well-being of humans by safeguarding against ailments such as diabetes, hypertension, and cancer (Emelike and Akusu, 2019).

The agricultural revolution and the adaptation of numerous tropical plants to regions outside of their natural range have muddied their classification, and little is known about what properly defines and distinguishes tropical fruits and vegetables from their temperate counterparts (Indiarto, 2020). Fernandes et al. (Fernandes et al., 2011) described crop classification according to size, acidity, seed type, and bearing. Included among alkaline crops are apples, bananas, peaches, cherries, persimmon, and litchi (Fernandes et al., 2011). Acidic crops include strawberry, orange, kiwi, pineapple, lemon, star fruit, and logan, whereas sub-acidic examples are mango, pear, blackberry, papaya, blueberry, cherimoya, and mulberry (Fernandes et al., 2011). Chakraborty et al. (Chakraborty et al., 2014) agreed and structured the classification of tropical fruits based on that of Fernandes. Sarkar et al. (Sarkar et al., 2018) reported classification system according to maturity stage by means of ethylene gas emission and respiration rate, including both climacteric and non-climacteric tropical produce (Sarkar et al., 2018). Tropical climacteric produce such as avocado, apple, pear, mango, papaya, broccoli, banana, kiwi, and tomato undergoes maturation in correlation with an escalation in their respiration rate and the release of ethylene gas (Indiarto, 2020), whereas tropical non-climacteric crops such as grape, berry, citrus, litchi, strawberry, raspberry, pumpkin, watermelon, cucumber, and pineapple do not undergo an elevation in their respiration rate as they reach maturity (Indiarto, 2020). The contrasting report of Retamales et al. (Retamales, 2011) centers around the production of temperate crops worldwide. In this report, apple, raspberry, pear, peach, kiwi, blueberry, strawberry and plum were considered as temperate fruits (Retamales, 2011). In addition, Benichou et al. (Benichou et al., 2018) have also classified temperate fruits as tree (apple, plum, pear and peach), vine (grape and kiwi), and small fruits such as raspberry, blueberry and currant (Benichou et al., 2018).

Papaya, pineapple, avocado, mango, and banana are considered to be major tropical fruits globally (Mukhametzyanov et al., 2022). According to a market review prediction for the years 2013 to 2022 by the Food and Agriculture Organization of the United Nations (FAO), the most exported tropical fruits globally from Central America and the Caribbean, South America and Asia, Africa, and others in millions of tons were papaya, pineapple, avocado and mango with 3.7, 3.2, 2.3, and 2.1, respectively (Altendorf, 2019). On the other hand, recent data have shown that global vegetable production increased by 68% between 2000 and 2021 (FAO, 2022). Because of the continuous and emergent demand for tropical fruits and vegetables worldwide, the present emphasis is on quality assurance in relation to end-user inclinations and commercial standards (Silva and Abud, 2017). The quality of tropical fruits and vegetables is characterized by both external and internal parameters (Jha and Matsuoka, 2000). External parameters namely color, defects, size and shape depend on not only the appearance of the product, but also on the standards set (Cubero et al., 2016), whereas internal parameters such as nutritional value, internal defects, flavor, and texture are subjective to physicochemical composition and climate change (Zainalabidin et al., 2019). The quality of fruits and vegetables influences consumer preference and is directly or indirectly linked with further value-addition and processing technologies (James et al., 2010).

Several studies have identified postharvest losses as the most prominent factor among the origins of crop quality deterioration (Porat et al., 2018; Etana, 2019; Ahmad et al., 2021). Adding to that, high temperature and relative humidity are mentioned in the biological and chemical degradation of produce freshness, which affects sweetness, flavor, weight, turgor, and nutritional value (Elik et al., 2019). However, past reports indicated that low-temperature cooling systems and edible coating materials can be used to maintain and monitor the quality of these crops (Mendy et al., 2019; Jodhani and Nataraj, 2021). Conventional methods relying on the quantification of different quality traits such as dry matter content, oil content, and moisture content have also been reported in the study of quality parameters of fruits and vegetables; however, these methods were found to be undesirable, destructive, time-consuming, and labor-intensive (Magwaza and Tesfay, 2015; Kyriacou and Rouphael, 2018). Therefore, the application of non-destructive bio-sensing methods as a promising alternative for evaluating the value of tropical produce has been adopted (Ndlovu et al., 2022; Okere et al., 2022).

Computer vision and popular pre-trained convolutional neural network (CNN) models have been used as recognition systems to sort and grade different fruits and vegetables, especially in supermarkets, regarding their variety and species (Dubey and Jalal, 2012). However, computer vision can only assess external quality attributes due to the lack of spectral information (Rahman and Cho, 2016; Bhargava and Bansal, 2021). Acoustic emission technology involves the mechanical destruction of produce when subjected to mechanical or thermal stimulus (Aboonajmi et al., 2015) and is not appropriate for all categories of fruits and vegetables (Adedeji et al, 2020 ). Extensive works have been published on the evaluation of fruits and vegetables by spectral measurements such as Fourier transform infrared (FTIR) spectroscopy (Egidio et al., 2009), Near-infrared (NIR), Raman spectroscopy (Pandiselvam et al., 2022), and hyperspectral imaging (HSI) (Wang and Zhai, 2018). Generally, these reports have concentrated on the utilization of spectral measurements for determining targeted quality parameters of a particular fruit or vegetable variety. For instance, visible and near-infrared spectroscopy was used to investigate the internal browning in mango fruits (Gabriëls et al., 2020). Ali et al. (Ali et al., 2023) investigated FTIR, NIR, and machine vision in the quality monitoring of pineapples. Metlenkin et al. (Metlenkin et al., 2022) distinguished Hass avocado fruits by defects using hyperspectral imaging (HSI). The question revolves around the practical utilization of these approaches and the challenges associated with improving data processing speed and in-line implementation (Cortés et al., 2019; Si et al., 2022). Quick hardware and software are required to fulfill the demands of swift analysis for extensive hyperspectral datasets (Xu et al., 2023) and machine learning algorithms, especially those relying on deep learning act as black boxes rather than using interpretability models for high-stakes decisions (Caceres-Hernandez et al., 2023).

The present review highlights the current advances in non-destructive spectral measurements for quality assessment, specifically for major tropical fruits and vegetables. The quality parameters of these tropical produces are covered first. The discussion on each of the spectral measurements, the tropical crops used, and the specific findings obtained from various studies, which are summarized in **Table 1**, follows and can deliver valuable information on the capabilities and efficiency of these techniques. In addition, the merits and demerits of each of these spectral measurements, which are presented in **Table 2**, will guide future researchers in selecting the proper evaluation method when evaluating the quality of tropical produces. To facilitate comprehension and quick understanding of key terminologies involved, the list of abbreviations and definitions contained in the paper is presented in **Table 3**.

**Table 1:** A comparison of the application of various non-destructive spectral measurements in the quality assessment of tropical fruits and vegetables.

Measurement	Tropical produce	Parameter	Data analysis	Performance (Accuracy)	Reference
FTIR, FTNIR	Pineapple	SSC TA PH	PCA	SD=0.17 SD=0.11 SD=0.13	(Egidio et al., 2009)
Vis–NIR, ML	Mango	Color	PLS, ANN	80%	(Gabriëls et al., 2020)
HSI	Avocado	Defects	PCA, PLS-DA, SIMCA	99.9%	(Metlenkin et al., 2022)
NIR	Mango	Firmness	PCA,MPLS	R^2 =^ 0.88 R^2 =^ 0.85	(Flores et al., 2008)
NIR	Papaya	Starch SSC	PLS	R=0.90 R=0.90	(Purwanto et al., 2015)
Vis–NIR	Pineapple	Nitrates	PLSR	R=0.95	(Srivichien et al., 2015)
HSI	Potato	SSC	PLSR	R^2^p=0.963	(Su and Sun, 2019)
FTIR	Banana	Maturity	PLS	R^2 =^ 0.83	(Zhang et al., 2021)
ATR-FTIR, ML	Banana	Ripening	PCA	96.0%	(Sinanoglou et al., 2023)
NIR	Avocado	Moisture content Dry matter	PLS	RPD= 2.00 RPD=2.13	(Olarewaju et al., 2016)
NIR	Mango	Maturity	MLR, PLS	Rc=0.74 Rv=0.68	(Jha et al., 2014)
NIR	Banana	TSS PH	PLS	R^2 =^ 0.81 R^2 =^ 0.69	(Ali et al., 2018)
NIR, HSI	Sweet potatoes	Variety identification	PLSDA	R^2 =^ 0.893	(Su et al., 2019)
NIR	Mango	Firmness	iPLSR	R^2^c = 0.75 R^2^p = 0.75	(Mishra et al., 2020)
Raman	Cassava	Starch adulteration	OC-SVM/SIMCA	86.9%	(Cardoso and Jesus Poppi, 2021)
Vis–NIR	Pineapple	Nitrate	PLSR	R= 0.95	(Srivichien et al., 2015)
HSI	Banana	SSC TA	PLS/iPLS/PLSDA	R^2 =^ 0.64 R^2 =^ 0.59	(Chu et al., 2022)
NIR–HSI	Pineapple	Water activity	PLSR	Rp= 0.72	(Aozora et al., 2022)
HSI, ML, DL	Papaya	Maturity	DCNN	F1 = 0.91	(Garillos-Manliguez and Chiang, 2021)
Raman	Sweet potato	Moisture and carotenoids	PLSR&PCA	R^2 =^ 0.90(hot air) R^2 =^ 0.88(microwave)	(Sebben et al., 2018)
Raman	Potato	Grading	PLSDA	≈100%	(Morey et al., 2020)
HSI	potato	Bruises	SVMM	87.88%	(Ye et al., 2018)
SWIR–HSI	Potato	Black spot	PLSDA	98.56%	(López-Maestresalas et al., 2016)
Raman	Mango	Carotenoids	–	R= 0.9618	(Bicanic et al., 2010)
Vis-NIR-HSI	Avocado	Nutrients (Fatty acids)	PLSR	R^2 =^ 0.79(flesh) R^2 =^ 0.62(skin)	(Kämper et al., 2020)
NIR–HSI	Mango	Defects	K-NN	97.95%	(Rivera et al., 2014)
HSI	Banana	Grading	CNN/MLP	98.45%	(Mesa and Chiang, 2021)

**Table 2:** Merits and demerits of non-destructive spectral measurements in the quality control of tropical fruits and vegetables.

Technique	Merits	Demerits	References
FTIR	No sample preparation.	Single beam and double beam for scattering device.	(Lan et al., 2020)
	Fast and easy to perform.	Difficulty in obtaining representative background.	
	Capability to measure many parameters at the same time.	Hard to read the interferogram if the Fourier transform is not performed first to generate the spectrum.	
	Good signal-to-noise ratio		
	Suitability for both quantitative and qualitative analyses.		
NIR	Real-time analysis.	Limited penetration depth.	(Srivichien et al., 2015), (Arendse et al., 2021)
	Can evaluate multiple components concurrently.	Time-consuming calibration procedure.	
	Fast acquisition of spectra.	Complex signal interpretation	
	Minimal sample preparation required.		
Raman	Vibrational and complementary.	Weak Raman scattering.	(Wang et al., 2021), (Li et al., 2016)
	Fast, Simple, sensitive, and selective technique.	Fluorescence interference.	
	Capability to monitor water-rich molecules.	Low reproducibility.	
	High spatial resolution.	Redundant data set. Costly Raman system.	
	Detects the spatial distribution of the molecules.	Relatively low operational speed	
HSI	Detect both spectral and spatial details.	Costly and complex data.	(Chandrasekaran et al., 2019), (Rajkumar et al., 2012)
	Concurrent assessment of many parameters.	Advanced hardware and software required.	
	Available in different algorithms.	Requires chemometrics techniques to extract relevant information.	

**Table 3:** List of abbreviations and acronyms used in the paper.

Abbreviation	Definition	Abbreviation	Definition
FTIR	Fourier transform infrared	CNN	Convolutional Neural Network
NIR	Near-infrared	TOF	Time of flight
HSI	Hyperspectral imaging	TSS	Total soluble solids
SSC	Soluble solid content	RGB–D imaging	Red, Green, Blue–Depth imaging
ASC	Added sugar content	PLS	Partial least squares
^0^C	Degrees Celsius	RMSE	Root mean square error
FAO	Food and Agriculture Organization	YOLO	You Only Look Once
R-CNN	Regions with convolutional neural networks	ATR	Attenuated total reflectance
L*, a*, and b*.	Lightness, redness or greenness, and yellowness	MLR	Multivariate linear regression
LED	Light-emitting diode	IR	Infrared region
R^2^	Determination coefficient	iPLSR	Interval partial least squares regression
TA	Total acidity	OC-SVM	One-class support vector machine
Vis–NIR	Visible–near-infrared spectroscopy	SIMCA	Soft independent modelling by class analogy
R	Coefficient of correlation	SERS	Surface-Enhanced Raman Spectroscopy
PLSR	Partial least squares regression	RMSEP	Root mean square error of prediction
R^2^P	Correlation of prediction	Rp	Coefficient of prediction
MIR	Mid-infrared	DT	Decision trees
FIR	Far-infrared	RNN	Recurrent neural network
ANN	Artificial neural network	PLSDA	Partial least square discriminant analysis
GA	Genetic algorithm	VGG	Visual Geometry Group
FL	Fuzzy logic	ResNet	Deep Residual Learning for Image Recognition
ANFIS	Adaptive neuro-fuzzy inference system	ResNeXt	Aggregated Residual Transformations for Deep Neural Networks
ML	Machine learning	DCNN	Deep convolutional neural network
DL	Deep learning	RPD	Residual predictive deviation
LDA	Linear discriminant analysis	F1 scores	Performance of Precision and recall
SVM	Support vector machine	MLP	Multilayer Perception
K-NN	K-nearest neighbors	PCA:	Principal component analysis
ELM	Extreme learning machine	MPLS:	Modified partial least square
RMSEC	Root mean square error of calibration	SD:	Standard deviation
Rc	Correlation coefficient for calibration	Rv	Correlation coefficient for validation

## 2 Quality inspection of Tropical fruits and vegetables

Quality inspection is the process of evaluating specific parameters of fruits and vegetables to ensure required quality standards (Phey et al., 2020). The intention of quality inspection is to detect any internal or external characteristics that can aid in identifying both standard quality parameters and defects or non-conformities that can affect the safety of fruits and vegetables or their usability in particular functions such as diets, trade, and industrial chains (Kirezieva et al., 2013).

### 2.1 External quality of tropical fruits and vegetables

The appearance of fruits and vegetables is a sensory attribute that directly influences the perceived worth of the produce for consumers (Zhang et al., 2014). The external quality of tropical crops is indicated by a number of factors, including size, shape, color, and external defects, as shown in **Table 4** (Ganiron, 2014). The size and shape are two complementary factors that differ depending on the variety of the plant and are both assessed in relation to market grading standards (Abbaszadeh et al., 2013). The size is determined by measuring area, perimeter, length, and width, which is more complex due to the morphological irregularities of tropical crops natural state (Cubero et al., 2011). Moreda et al. (Moreda et al., 2009) described some non-invasive systems for assessing the size of fruits and vegetables. The systems are based on (1) measuring the volume of the gap between the fruit and the outer casing of an embracing gauge; (2) measuring the distance between a radiation source and the fruit contour, where this distance is computed from the time of flight (TOF) of the propagated waves; (3) light obstruction by barriers or blockades of light; (4) 2D and 3D machine vision systems (Moreda et al., 2009).

**Table 4:** The external quality parameters of tropical fruits and vegetables.

External quality	Indicators		References
Size	Area, perimeter, length, and width		(Cubero et al., 2011), (Sanchez et al., 2020)
Shape	Mass, volume, spherical coefficient, density, and geometric mean diameter		(Cubero et al., 2011), (Golmohammadi and Afkari-Sayyah, 2013)
Color	Maturity, uniformity, and intensity		(Yahaya et al, 2017), (Ali et al., 2022)
External defects	Bruising, crushing, shriveling, and wilting		(Ali et al., 2023), (Raj and Suji, 2019)

Wang et al. (Wang et al., 2017) evaluated mango size by RGB–D (depth) imaging and time-of-flight camera imaging system. The camera-to-fruit distance was determined using three methods for fruit sizing from images: stereo vision camera, RGB–D camera and a time-of-flight laser rangefinder (Wang et al., 2017). The obtained length and width values were good with RMSE of 4.9mm and 4.3mm respectively. It is cost-effective and simple to use; however, it pertains non-occluded fruit only and cannot be utilized in direct sunlight (Wang et al., 2017). Neupane et al. (Neupane et al., 2022) replicated the work of Wang by suggesting the use of partly occluded fruit. To obtain the linear length of the fruits, bounding box dimensions of an instance segmentation model (Mask R-CNN) was applied to canopy images (Neupane et al., 2022). The findings were good with RMSE values of 4.7 mm and 5.1 mm for Honey Gold and Keitt mango varieties, respectively (Neupane et al., 2022). Sanchez et al. (Sanchez et al., 2020) investigated spectroscopic and depth imaging techniques combined with machine vision to estimate the length, width, thickness, and volume of sweet potato and potato. When the correct size group was graded, the method had a high accuracy of 90% (Sanchez et al., 2020).

Color is an external quality trait that depends on the maturity of produce and is subjective to internal features such as taste, perception, and pleasantness of fruits and vegetables (Yahaya et al, 2017). Calorimeters evaluate color by measuring the typical surface area of the product and detects the color space values L*, a*, and b* which are based on the human color perception theory (Aguilar-Hernández et al., 2021). The capability of infrared thermal imaging approaches was investigated in the measurement of pineapple color. In this investigation, the L*, a*, and b* mean values for calorimeter increased by (P < 0.05) (Ali et al., 2022). The optical fiber sensors mounted with RGB LEDs were also used to evaluate the color of mangoes, giving R^2 =^ 0.879 (Yahaya et al., 2011).

External defects include the evidence of rot, bruising, crushing, shriveling, and wilting due to water loss which impact market value and the price of the fruits and vegetables (Raj and Suji, 2019). These defects can be recognized and monitored through the appearance of the crop by qualified personnel relying on subjective evaluation, which may result in human errors (Ali et al., 2023). Sahu et al. (Sahu and Potdar, 2017) proposed a digital image analysis algorithm for detecting exterior defects in mango fruit. Surface defects such as scars and black patches were used to detect defective mango fruits, and were recognized by extracting the contours of damaged areas (Sahu and Potdar, 2017). The damaged area was then filled to identify its location in the image as the basis for discrimination. Sahu and colleagues achieved good accuracy but advocated the use of optimal and adaptive threshold approaches for segmenting mango fruits from image backgrounds (Sahu and Potdar, 2017).

### 2.2 Internal quality of tropical fruits and vegetables

The internal qualities of fruits and vegetables are also termed hidden qualities and are determined by texture, nutrients, internal defects, and flavor, as presented in **Table 5** (Shewfelt, 2014). Different fruits and vegetables usually have different textures, which are characterized by their firmness, crispness, and crunchiness (Fillion and Kilcast, 2002). The assessment of fruit and vegetable firmness, a vital quality characteristic related to texture, can be achieved through sensory measurements (Magwaza and Opara, 2015). The texture is measured with a penetrometer by putting a probe tip installed on the texture analyzer into fruit tissue at a specific speed and depth so as to exert the most force (Ali et al., 2017). Uarrota et al. (Uarrota and Pedreschi, 2022) used a non-destructive texture analyzer to determine the firmness of avocado under different storage conditions. Enough data were required to construct the best model allowing an extension to the model firmness of avocado (Uarrota and Pedreschi, 2022). Kasim et al. (Kasim et al., 2021) compared laboratory-based (305-1713 nm) and portable-based (740-1070 nm) NIR spectrometers to determine mango firmness (Kasim et al., 2021). The results showed that portable and laboratory-based NIR instruments performed similar in respect of R^2^p. Compared to the laboratory-based instrument, the RMSEP of the portable NIR was higher (Kasim et al., 2021).

**Table 5:** The internal quality parameters of tropical fruits and vegetables.

Internal quality	Indicator	References
Texture	Firmness, crispness, and juiciness	(Fillion and Kilcast, 2002), (Magwaza and Opara, 2015)
Nutrients	Chemical compositions (vitamins, sugars, proteins, and functional properties)	(Leiva-Valenzuela et al., 2013), (Aziz et al., 2021)
Internal defect	Internal cavity, water core, and rot	(Yahaya et al, 2017), (Ruiz-Altisent et al., 2010)
Flavor	Sweetness, sourness, saltiness, and bitterness	(Yahaya et al, 2017), (Zhu et al., 2020)

Nutritional value, such as the sugar content related with vitamins and minerals, comprises the main constituents of soluble solids content (SSC), total soluble solids (TSS), and total acidity (TA) (Leiva-Valenzuela et al., 2013). Aziz et al. (Aziz et al., 2021) evaluated the relationship between TSS and the capacitance of papaya using capacitance-sensing techniques (Aziz et al., 2021). A refractometer was used as part of a destructive technique to predict the reference values of moisture and TSS content. Capacitive sensing was then tested as non-destructive approach for the evaluation of output voltage and capacitance of papaya (Aziz et al., 2021). Aziz observed a good correlation between destructive and non-destructive techniques, with R^2^ of 0.9434 and 0.9177 for moisture and TSS content, respectively (Aziz et al., 2021). The usefulness of NIR spectroscopy was demonstrated in the determination of starch and soluble solid contents of papaya (Purwanto et al., 2015). Srivichien and colleagues tested the nitrates in pineapples using Vis–NIR (600-1200 nm) spectroscopy, yielding an R value of 0.95 (Srivichien et al., 2015). However, due to the big size and the change in nitrate levels, many scans were needed on different areas of pineapple (Srivichien et al., 2015). In the study to predict starch content of sweet potatoes and potatoes, hyperspectral imaging was applied by Su et al. (Su and Sun, 2019). Su developed partial least squares regression (PLSR) models at full-wavelength referring to spectral profiles and observed reference values, resulting in a high accuracy and an R^2^P of 0.963 (Su and Sun, 2019).

Internal defects are detected as internal injury such as rot and water core inside the flesh of the fruits and vegetables due to postharvest problems(Ruiz-Altisent et al., 2010). Flavor or taste is defined by the sugar (sweetness), acidity (sourness), bitterness, and saltiness perceived by the tongue and nose (Zhu et al., 2020). It is, therefore, measured subjectively through oral testing or smelling, or by the conventional technical quantification of compounds such as liquid and gas chromatography (Yahaya et al, 2017). Korean universities conducted research on the taste and odor properties of broccoli using electronic sensors (Hong et al., 2022). For electronic tongue analysis, thermal processing boosted sourness and umami tastes while decreasing saltiness, sweetness, and bitterness (Hong et al., 2022). Therefore, the capability of non-destructive spectral measurement methods to assess inside parameters is important to maintain the flesh quality of tropical fruits and vegetables.

## 3 Non-destructive spectral measurements for the quality evaluation of tropical fruits and vegetables

**Figure 1:**
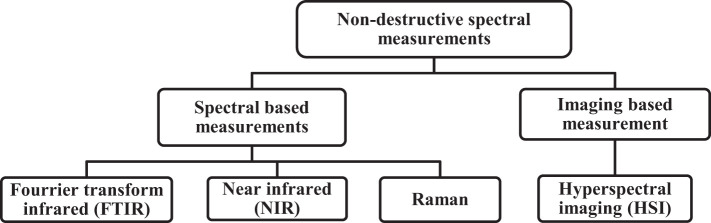
The schematic diagram of commonly used non-destructive spectral measurements.

Non-destructive techniques for quality monitoring of tropical fruits and vegetables refer to the process of inspecting their external and internal properties without causing damage or changing their physical and internal status (El-Mesery et al., 2019). The potential for employing spectral measurement approaches in the quality control of fruits and vegetables is growing enormously (Escárate et al., 2022). The reason is that these approaches are non-destructive, fast and accurate, capable for both quantitative and qualitative analysis, thereby requiring minimal sample preparation (Cozzolino, 2022). We divided non-destructive spectral measurements into two categories: (1) spectral-based approaches (FTIR, NIR, and Raman spectroscopy) and (2) imaging-based approaches (HSI), as shown in **Figure 1**.

### 3.1 Spectral-based approaches

**Figure 2:**
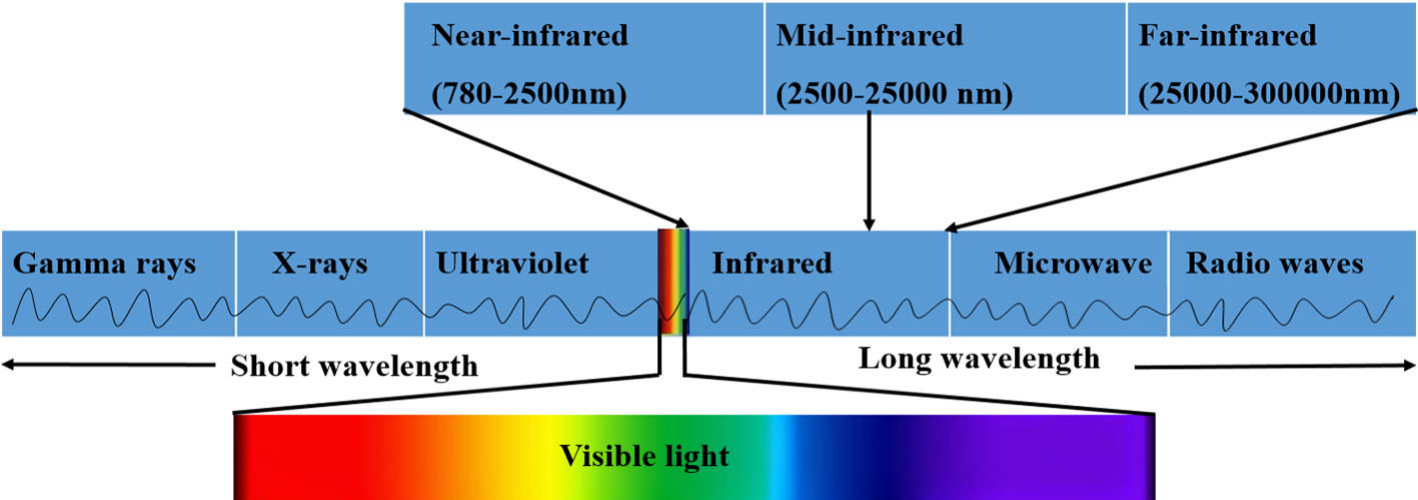
Modified diagram showing the infrared regions of the electromagnetic spectrum (Yeap and Hirasawa, 2019), (Aboud et al., 2019).

Spectral measurement refers to effective techniques used to study the quality parameters of various agricultural materials including tropical fruits and vegetables by investigating light, sound, or particles that are emitted, absorbed, or scattered during measurement (Pathare and Rahman, 2022). Spectroscopic techniques based on FTIR, NIR, and Raman have been successful and popular in the detection of quality parameters of fruits and vegetables (Dasenaki and Thomaidis, 2019). Various research works have used spectral techniques focusing on fruits and vegetables, such as in the fast determination of the sugar and acid composition of citrus (Clark, 2016), assessment of primary sugars and amino acids in raw potato tubers (Ayvaz et al., 2015), and determination of nutrients and moisture content of fruits and vegetables (Sirisomboon, 2018). Quality parameters of tropical crops can be assessed by one of—or a sequence of—the above complementary techniques, which are distinguished depending on the infrared region (IR) they occupy and the molecular vibrations they detect (Bureau et al., 2019). The infrared region of the electromagnetic spectrum, presented in **Figure 2**, is separated into three sections, namely near-infrared (NIR), mid-infrared (MIR), and far-infrared (FIR) (Yeap and Hirasawa, 2019). Mango maturity has been predicted using the near-infrared (NIR) spectral region of 1200-2200 nm (Jha et al., 2014). The mid-infrared (MIR) spectral range of from 2500 to 25000 nm has been used in the prediction of banana maturity and geographical origin by Zhang et al. (Zhang et al., 2021), and in the measurement of soluble solids, total acids, and total anthocyanin in berries (Clark et al., 2018). Far-infrared (FIR) ranges have often been reported to be between 25000 and 300000 nm (Larkin, 2017). However, FIR applications are not clearly defined and are limited due to challenges in developing FIR instrumentation; furthermore, the band assignments of low-frequency vibrational modes are not straightforward (Ozaki, 2021). These spectral ranges are based on their relationship to the visible spectrum, which falls between 380 and 780 nm (Su and Sun, 2018).

#### 3.1.1 Fourier transform infrared spectroscopy

**Figure 3:**
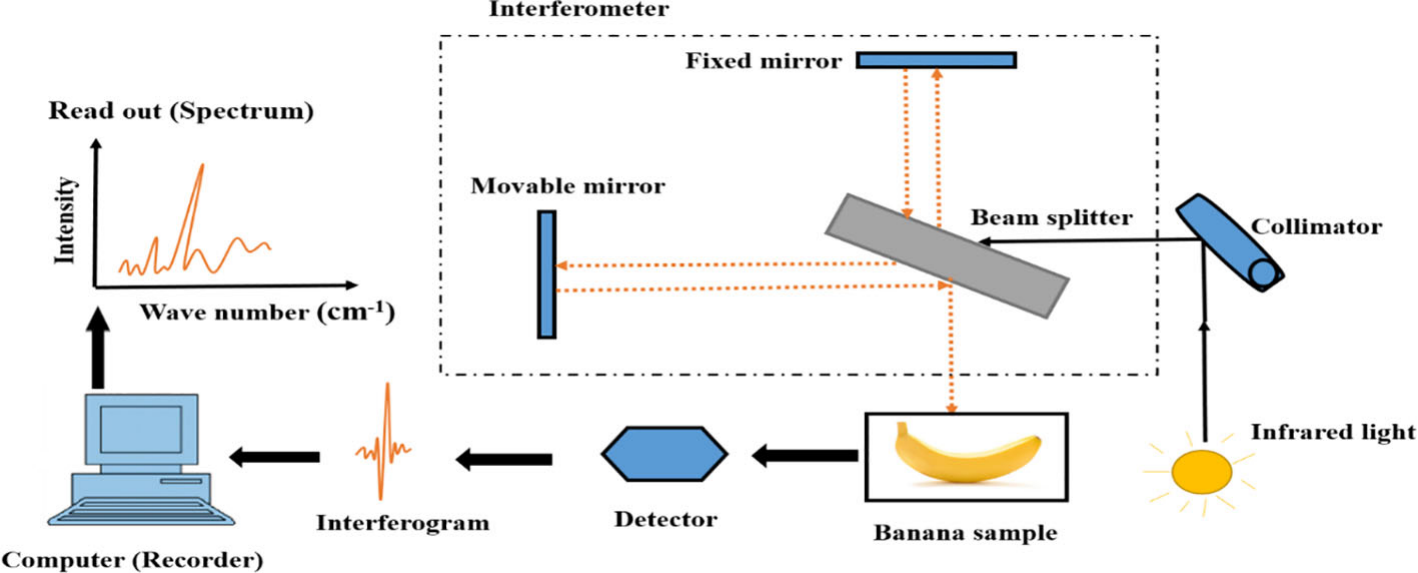
Modified diagram of FTIR spectroscopy taking banana as sample (Patrizi and Cumis, 2019).

FTIR is a form of vibrational spectroscopy that uses light interference to identify the chemical composition of scanned samples by producing infrared absorption or emission spectra (Larkin, 2017). On the electromagnetic spectrum, FTIR operates in the MIR region (2500 to 25000nm) and generates fruit or vegetable chemical profile by capturing the principle vibrational and rotational stretching modes of molecules (Lohumi et al., 2015). FTIR spectroscopy comprises of an infrared light source, interferometer, sample, and detector, shown in **Figure 3**. The principal part is the interferometer which is made up of three components: the beam splitter, collimator, and the two mirror (fixed and movable mirror) (Patrizi and Cumis, 2019). When the radiation from the light source passes through the collimator, strikes the beam splitter which ideally divide it into two beams. The first beam hits the static mirror, and is reflected back; while the second hits the movable mirror where it enters through the sample toward the detector (Blum and Harald, 2012).

The FTIR associated with attenuated total reflection (ATR-FTIR) has recently gained importance (Chan and Kazarian, 2016). The ATR works under the principle of total internal reflectance where infrared light interacts with the sample of high refractive index only at the point where infrared light is reflected (Ryu et al., 2021). Unlike transmission methods, the ATR-FTIR technique can be used to study solid, liquid, and paste samples with minimal sample preparation (Glassford et al., 2013).The combination of ATR-FTIR and chemometrics was promising in the assessment of added sugar content, (ASC), total soluble solids (TSS) and real juice content (RJC) of fresh and commercial mango juice (Jha and Gunasekaran, 2010). PLS and MLR models resulted into accuracy of 0.99 and 0.98 respectively (Jha and Gunasekaran, 2010). Canteri et al. (Canteri et al., 2019) have used ATR-FTIR to evaluate the cell wall compositions of 29 species of fruits and vegetables as freeze-dried powders and alcohol-insoluble solids. The results were accurate, with determination coefficient R^2^ ≥ 0.9 (Canteri et al., 2019). Recently, Sinanoglou et al. (Sinanoglou et al., 2023) conducted the evaluation of both peel and fresh banana ripening stage by ATR-FTIR, along with image analysis, discriminant and statistical analysis (Sinanoglou et al., 2023). The computed features were accurate enough to separate ripening stages; however, monitoring of the banana ripening process was highly reliant on the instrument employed for image analysis such as digital cameras, smartphones, and electronic noses (Sinanoglou et al., 2023).

#### 3.1.2 Near-Infrared spectroscopy

**Figure 4:**
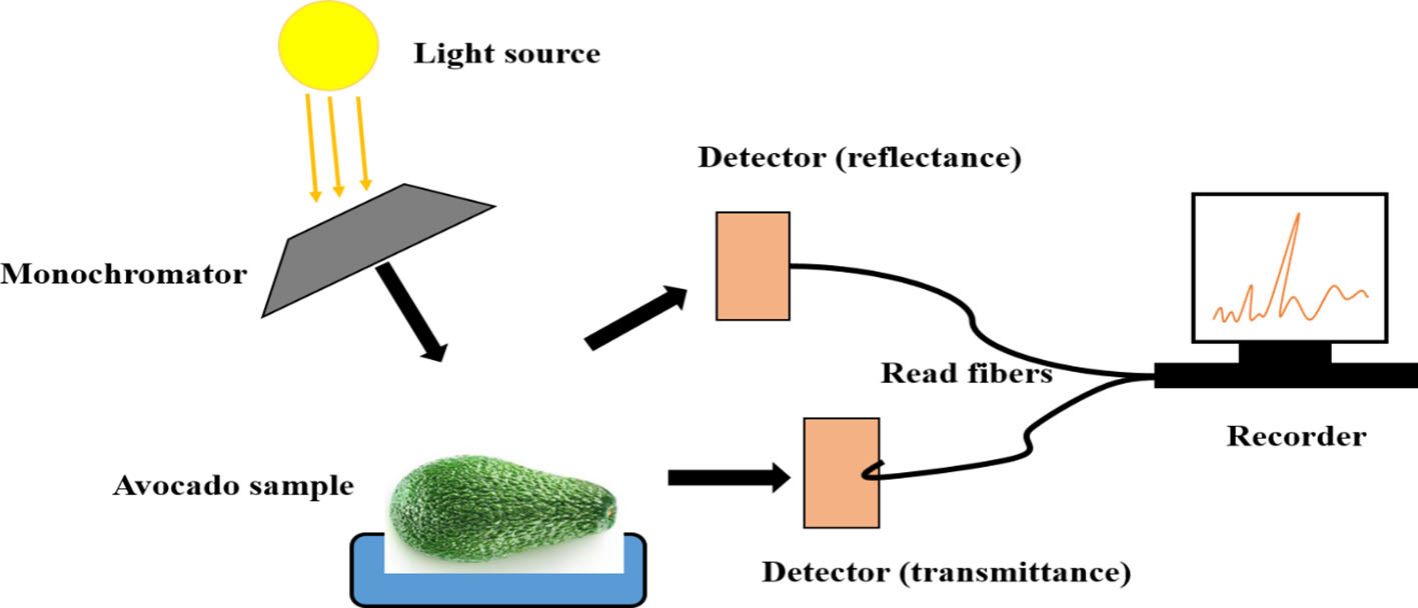
Modified diagram of NIR spectroscopy, taking avocado as sample (Chandrasekaran et al., 2019).

NIR is used to rapidly ascertain the chemical constitution of materials according to overtones and harmonic or combination bands of specific functional groups (Kusumaningrum et al., 2018). Those overtones and combinations of vibrational bands characterized by C–H, O–H, and N–H are gained by NIR in the wavelength region of 780-2500nm (Ozaki et al., 2006). Tsuchikawa et al. (Tsuchikawa et al., 2022) described NIR as a spectroscopic method that is suitable for samples of high water content, including fruits and vegetables (Tsuchikawa et al., 2022). NIR spectroscopy consists of a light source, sample accessory, monochromator (grating), detector, and optical components such as lenses and optical fibers, as shown in **Figure 4** (Lee et al., 2011).

The illumination of NIR light to the sample occurs in three ways: reflectance, interactance and transmittance (Wang et al., 2014). According to Hong and colleagues, reflectance employs high light energy, has no contact with the fruit surface, and the source and sensor are placed at a specified angle (Hong and Chia, 2021). Specular reflectance and diffuse reflectance are two types of reflectance measurement. Specular reflectance, which occurs when the incident and reflected angles are same, detects nothing from the inside part of the fruit (Hong and Chia, 2021); While the capacity of diffuse reflectance to constrain light dispersion into solid samples allows the acquisition of interior fruit information (Tang et al., 2022). Mango TSS, firmness, TA, and ripeness index (RPI) were effectively measured by NIR diffuse reflectance, with R^2^ of 0.9; 0.82; 0.74; and 0.8, respectively. The effect of changes in physicochemical properties of mango during ripening, on the other hand was highlighted (Rungpichayapichet et al., 2016). Kusumiyati et al. (Kusumiyati and Suhandy, 2021) also evaluated TSS and Vitamin C using the same fruit and NIR spectra acquisition mode. The diffuse reflectance spectra were documented and found to be in relation with TSS, vitamin C (Kusumiyati and Suhandy, 2021).

Delwiche et al. (Delwiche et al., 2008) demonstrated the use of near infrared interactance (750-1088nm) to determine mango ripeness, SSC and other sugars. The mango sample was placed in contact with the probe in which the top of mango upwardly points the probe. The R^2^ was 0.77; 0.75; 0.67; and 0.70 for SSC, sucrose, glucose, and fructose, respectively. Sugars such as sucrose indicates mango sweetness, fructose and glucose increases during ripening while acidity decreases (Delwiche et al., 2008). Transmission mode in which the light source and sensor are opposite to each other, employs low light intensity to reflect the inner parameters and is performed with no contact on the fruit (Nicolaï et al., 2007). Transmission might be done partially or fully. Though, the difference between partial transmission and diffuse reflectance remains undetermined since both evaluate the radiation that partly enters the sample and diffusely reproduced to the sensor (Hong and Chia, 2021). The fruit with large seed such as mango was reported to be hard to measure in the full transmission due the low signal to noise ratio (Greensill and Walsh, 2000). Subedi at al. (Subedi and Walsh, 2011) detected the TSS and DM of mesocarp tissue of banana and mango by partial transmittance. Mango DM gave R^2^cv =0.75 while banana performance negatively influenced by the thickness of the peel. The TSS results on mango was good in ripe and poor in ripening stage with R^2^cv > 0.75 and R^2^p < 0.75 respectively. The results were consistent with those of Rungpichayapichet et al. (Rungpichayapichet et al., 2016) and were found to be caused by the physiological factors of Mango, banana, and other tropical fruits which can change their starch content as they ripe (Subedi and Walsh, 2011).

Several studies have highlighted the potentials of NIR spectroscopy to monitor the internal and external characteristics of tropical fruits and vegetables, including the following: maturity prediction of avocado and mango (Olarewaju et al., 2016; S. N. Jha et al., 2014), total soluble solids and pH of banana (Ali et al., 2018), and variety identification in sweet potatoes (Su et al., 2019). However, the irregular thick skin of pineapple and chemical complexity of large seeded mango was the main difficulty to Guthrie et al. (Guthrie and Walsh, 1997) in the measurement of SSC by NIR reflectance (760-2500nm). The penetration depth of NIR light into a thick-rind avocado 38 mm in diameter and 10 mm in thickness was investigated for the maturity evaluation of avocado using an NIR spectrometer (800–2400 nm) (Olarewaju et al., 2016). The models for estimating oil content, were acceptable, however were not accurate, with an RPD value of less than 1.0 and an R^2^ value of 0.58 (Olarewaju et al., 2016). Arendse et al. (Arendse et al., 2018) informed the limited accuracy of NIR for internal quality assessment of fruits and vegetables with thick rinds such as banana, avocado and pineapple due to inadequate penetration depth (Arendse et al., 2018). Therefore, future studies can consider the appropriate selection of NIR optical geometry and wavelength range to improve the prediction accuracy of thick rind tropical crops (Pratiwi et al., 2023).

NIR spectral data inevitably holds overlay information of numerous organic compounds at global wavelengths, making the use of global spectroscopic regions problematic rather than specific wave bands (Lin and Yibin, 2009). Therefore, a combination of algorithms and chemometrics with NIR spectroscopy is now being used to meet this demand, balance data redundancy and complexity, and collect spectral information (Guan et al., 2019; Yang et al., 2021). Portable NIR spectroscopy was used to assess mango firmness during ripening (400–1130 nm) (Mishra et al., 2020). Pre-processing was done Savitzky–Golay filter, and iPLSR model was found to provide better predictive modeling, with an R^2^p of 0.75 and an RMSEC of 5.92 Hz^2^g^2/3^ compared to the standard PLSR model, which had an R^2^p of 0.67 and an RMSEC of 6.88 Hz^2^g^2/3^. For the firmness in mango fruit, spectral intervals 743-770 nm and 870-905 nm were found to be the accurate predictors (Mishra et al., 2020).

#### 3.1.3 Raman spectroscopy

**Figure 5:**
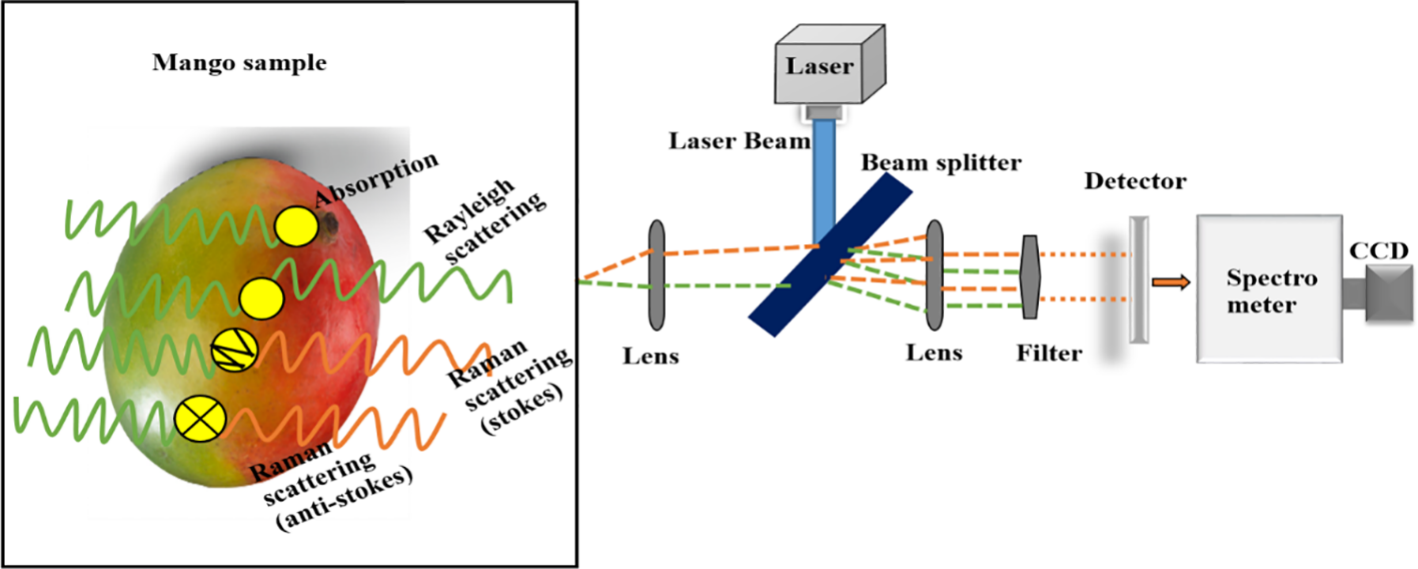
Modified diagram of Raman spectroscopy, taking mango as sample (Lohumi et al., 2015).

Raman is another form of vibrational spectroscopy that uses laser beams to interact with materials and operates in the infrared region of the electromagnetic spectrum from 2500 to 25000 nm (Siesler et al., 2008). Though Raman and MIR spectroscopy methods use high levels of energy to detect molecular vibrations, Raman spectroscopy excels at equal vibrations of nonpolar sets, while MIR spectroscopy excels at the unequal vibrations of polar sets (Campanella et al., 2021). Raman spectroscopy consists of a monochromatic laser, wavelength separator, and a detector, as presented in **Figure 5** (Qin et al., 2019). When the laser beam illuminates the sample, the photons that constitute the light are absorbed, transmitted, or scattered by the sample in different directions before reaching the detector (Larkin, 2017). Absorption and transmission are linked with the infrared spectra (IR), while scattering is associated with the Raman spectra (Jones et al., 2019). Rostron et al. (Rostron et al., 2016) defined scattered photons in two different ways namely Rayleigh (elastic) scattering and Raman (inelastic) scattering (Larkin, 2017). Rayleigh (elastic) scattering occurs when the photons scattered are equal to those illuminated to the sample; while Raman (inelastic) scattering is due to the transfer of energy between photons and the sample under testing (Lu, 2017).

Raman spectroscopy is suitable for investigating carotenoids in various plants, including carrots (Lawaetz et al., 2016), tomatoes (Hara et al., 2018), plant cells (Baranska et al., 2011), and mango (Bicanic et al., 2010). Furthermore, Raman has been applied as a clean and fast approach to assess cassava starch adulteration (Cardoso and Jesus Poppi, 2021). Two chemometrics models, namely one-class support vector machines (OC-SVMs) and soft independent modelling by class analogy (SIMCA), were used and compared statistically. The OC-SVM results outperform those of SIMCA, with an accuracy of 86.9% (Cardoso and Jesus Poppi, 2021). Surface-enhanced Raman spectroscopy (SERS) was used as a method that applies Raman spectroscopy in conjunction with nanotechnology for the fast analysis of pesticide residues in mango (Pham et al., 2022). SERS results were good indicating that the residues in mango sample were in the suitable range (Pham et al., 2022). Morey et al. (Morey et al., 2020) used spatially offset Raman spectroscopy for potato varieties quality categorization and prediction of tuber cultivation source. This approach is fast since it can be used directly after potato harvesting (Morey et al., 2020).

### 3.2 Imaging-based approaches

Spectral imaging techniques are among the most effective detection methods because of their potential to obtain both spectral and spatial dimensions of produce simultaneously during measurement (Liu et al., 2017). Regarding spatial dimensions, external attributes such as size, shape, appearance, and color can be evaluated, while with spectral analysis, internal features such as chemical composition can be measured (Pu et al., 2015). A number of imaging techniques use two-dimensional geometry according to the fusion and luminance of color maps (Lu et al., 2014), while others involve the use of three-dimensional sensors such as RGB and hyperspectral images (Barnea et al., 2016) to provide a high fruit and vegetable recognition accuracy (Nyarko et al., 2018).

#### 3.2.1 Hyperspectral imaging techniques

**Figure 6:**
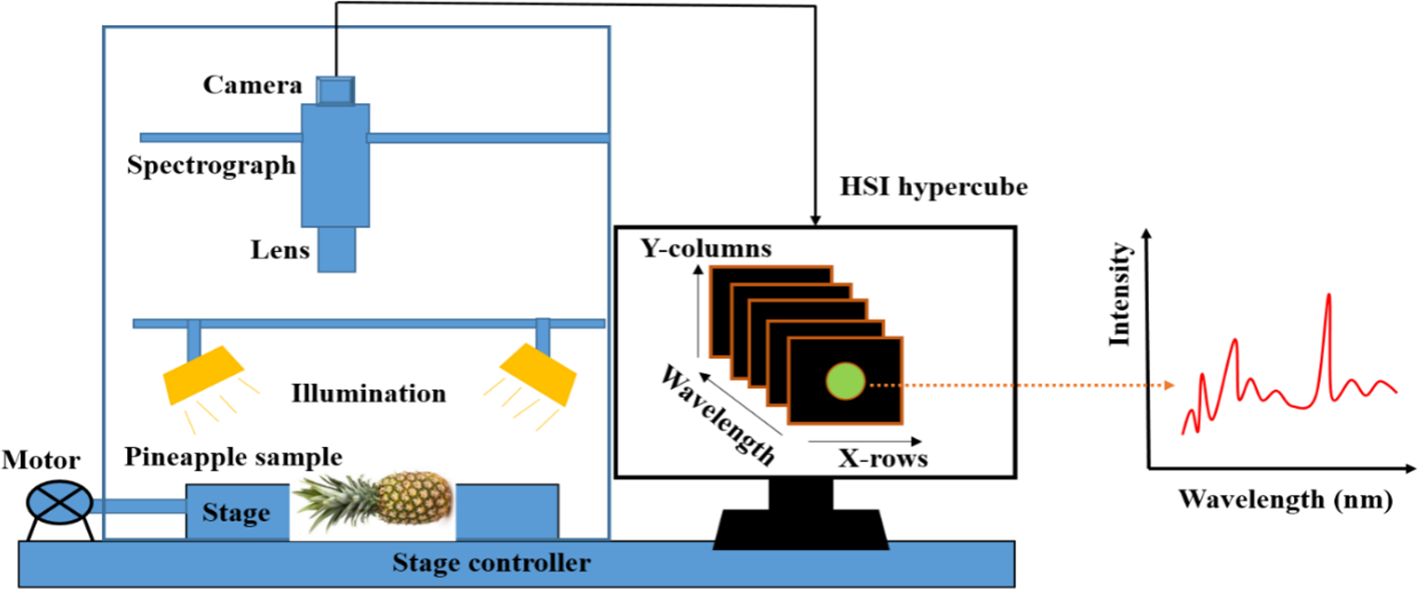
Modified diagram of Hyperspectral imaging, taking pineapple as sample (Li et al., 2018).

In agriculture and food systems, hyperspectral imaging is a powerful system that joins two aspects of imaging and spectroscopy to attain a three-dimensional (3D) hypercube data form and analyzes a broad spectrum at each pixel instead of assigning only main RGB colors (red, green, and blue) (Khan et al., 2021). The hypercube consists of 3D images characterized by 2D spatial and 1D spectral dimension or wavelength (Tang et al., 2022). Hyperspectral imaging employs more than ten contiguous wavelengths or narrow bands in which each pixel has a full continuous spectrum (Elmasry et al., 2019). To take sample images, the hyperspectral imaging set up can be in the reflectance, transmittance, and interactance which differs in their lighting configuration during crops measurements (Pan et al., 2017). The reflectance geometry is appropriate for assessing the external quality of products, whereas the transmittance performs better in measuring the internal components in relatively translucent membranes (Li et al., 2018). The HSI system comprises of four main components: (1) an imaging unit, (2) illumination (light source), (3) a sample stage, and (4) a computer, as presented in **Figure 6** (Pu et al., 2015). The light source is divided into illumination and excitation sources for spectral imaging applications. Broadband lights are commonly used as an illumination source for reflectance and transmittance, whereas narrowband lights are for the excitation source (Qin et al., 2013). The lighting devices produce light that illuminates the sample. The camera transports chemical information as well as light from the light source. The wavelength dispersion device, which can be a grating or a prism, divides the light into different wavelengths and directs the dispersed light to the sensor (Wu and Sun, 2013). Aozora et al. (Aozora et al., 2022) studied the efficiency of hyperspectral imaging (935–1720 nm) in the evaluation of water activity in dehydrated pineapple. The accuracy of the tested model showed good accuracy, with 0.72 and 0.0054 for Rp of and RMSEP respectively (Aozora et al., 2022).

##### 3.2.1.1 Hyperspectral imaging Image generation modes

HSI generates image in three ways: whisk broom (point scanner), push broom (line scanner), and tunable filter (area scanner) (ElMasry and Sun, 2010). The point scan excites only a single spot on the object’s surface and the single pixel is recorded. The spectrum is taken at both positions by moving the sample symmetrically in two spatial dimensions, in order to get the full HSI image (Qin, 2012). However, to obtain good results this technique involves double scanning of the sample and hardware relocation which takes a lot of time to complete the measurement (Qin, 2012). The line scanner excites a line on the object and records the whole line of an image using a 2D dispersing element and 2D detector array. The object is moved line by line and the whole set of spatial–spectral data is gained. This approach has a higher acquisition rate but lower sectioning ability (Qin, 2010). The area scan employs spectral scanning techniques to stimulate the broad area on the surface of the fruit or vegetable, which is held fixed and a scan with full spatial information is achieved consecutively across the entire spectral range. This method is appropriate for applications where sample mobility is not necessary (Lu et al., 2017).

The hyperspectral imaging together with chemometrics models is an appealing option for dealing with large sets of complex, high-dimensional data (Lorente et al., 2012). Chu et al. (Chu et al., 2022) confirmed the efficacy of the HSI reflectance (386-1016 nm) wavelength region in combination with variable selection algorithms and chemometrics for predicting green banana maturity level and characterization of banana quality during maturation (Chu et al., 2022). The line scanning approach was adopted and the calibration models used were partial least squares (PLS) and interval PLS methods (Chu et al., 2022). These models obtained acceptable values R^2 =^ 0.64 and 0.59 for SSC and TA, respectively, whereas the models for chlorophyll and ΔE* were suitable only for sample screening with R^2 =^ 0.34 and 0.30, respectively (Chu et al., 2022). Chu reported the inclusion of more samples and different cultivars of banana for model improvement (Chu et al., 2022). Kämper et al. (Kämper et al., 2020) used Vis–NIR–HSI to measure nutrients in avocado fruit. PLSR was used to obtain the ratio of unsaturated to saturated fatty acids in avocado fruit with (R^2 =^ 0.79, RPD = 2.06) and (R^2 =^ 0.62, RPD = 1.48) for flesh images and skin images respectively (Kämper et al., 2020). The robust models for flesh images were R^2 =^ 0.67; 0.61; and 0.53, of oleic-to-linoleic acid ratio, boron (B) and calcium concentration (Ca) respectively, while for skin images was R^2 =^ 0.60 of boron (Kämper et al., 2020).

## 4 Advancement in non-destructive spectral measurements for tropical fruit and vegetable quality assessment

The rapid advancement of technology in the agricultural field has resulted in the combination of artificial intelligence with non-destructive spectral measurements for fruits and vegetables quality measurement (Hasanzadeh et al., 2022). Artificial intelligence models such as artificial neural networks (ANNs), genetic algorithms (GAs), fuzzy logic (FL), and adaptive neuro-fuzzy inference system (ANFIS) can assess multiple characteristics simultaneously (Homayoonfal et al., 2022). Salehi reviewed development of models used in the determination of fruits and vegetables quality (Salehi, 2020). ANNs, GAs, FL, and ANFIS detected defects, moisture content, and chilling injury of oranges, cherries, pomegranates, apples, peaches, avocados, button mushrooms, tomatoes, and potatoes (Salehi, 2020). Despite the fact that these models are typically constrained by normality, linearity, homogeneity, and variable independence, the ANFIS model outperforms others and can be successfully used in relevant research (Salehi, 2020).

Machine learning (ML) is a branch of artificial intelligence and an integral part of the development of many sensing technologies that are responsible for information retrieval, signal processing, and data analysis (Li et al., 2021). In recent decades, traditional algorithms such as linear discriminant analysis (LDA), support vector machines (SVMs), K-nearest neighbors (K-NN), naïve Bayes, extreme learning machines (ELMs), decision trees (DTs), and K-means clustering have been deployed (Fadchar and Dela Cruz, 2020). For instance, Rivera et al. (Rivera et al., 2014) used NIR–HSI and machine learning for the early detection of mechanical damage in mango. LDA, K-NN, naïve Bayes, ELMs, and DTs were used for categorization. Bayes failed, however (K-NN, ELM, DT, and LDA Title altered) results was more than 90%. The highest performance, achieved by K-NN, was 97.9% (Rivera et al., 2014).

The evolution of deep learning (DL) as a breakthrough machine learning method has been trending since 2017 due to the manual feature extraction of traditional machine learning methods (Yang and Xu, 2021) and limited performance of chemometrics models, such as spectral variability caused by sample and spectrometer heterogeneity, changing environmental conditions, and infrared spectral data with high noise, which hinder feature extraction using chemometrics models (Zhang et al., 2021). Deep learning is a subset of machine learning that use many neural network layers to extract complex feature representations with numerous levels of abstraction (Lecun et al., 2015). According to Kamilaris et al. (Kamilaris and Prenafeta-Boldú, 2018), convolutional neural network (CNN) and recurrent neural network (RNN) have been implemented for crop-type classification, counting produces, and locating their placement in the image using bounding boxes (Kamilaris and Prenafeta-Boldú, 2018). However, the RNN was found to perform better than the CNN because it considers not only space but also the time which helps to capture the time dimension (Kamilaris and Prenafeta-Boldú, 2018). Deep learning and machine learning technology-based spectral analysis has been used in the classification of three types of fruits (apple, lemon, and mango) by type of damage, type of goods, and whether the sample is raw in market, supermarket, wholesaler, and retailer applications (Bobde et al., 2021).

Garillos-Manliguez et al. (Garillos-Manliguez and Chiang, 2021) estimated six maturity stages of papaya fruit, from the unripe stage to the overripe stage, by feature concatenation of data obtained from visible light and HSI imaging (Garillos-Manliguez and Chiang, 2021). AlexNet, VGG16, VGG19, ResNet50, ResNeXt50, MobileNet, and MobileNetV2 architectures was then modified to apply multimodal data cubes made of RGB and hyperspectral data (Garillos-Manliguez and Chiang, 2021). Regarding classification of the six stages, these multimodal variations can reach F1 scores of up to 0.90 and a 1.45% top-2 error rate. However, due to the small size of the images and the great depth of the CNNs, resulting in highly tightly tuned training variables, overfitting may arise. On the other hand, increasing image size results in insufficient memory faults (Garillos-Manliguez and Chiang, 2021).

Banana fruit was graded by Mesa et al. (Mesa and Chiang, 2021) using multi-input deep learning model with RGB and HSI. These models were able to categorize tier-based bananas by 98.45% and an F1 score of 0.97 with only few samples (Mesa and Chiang, 2021). However, this technique is expensive and time consuming due to the use of two cameras. The next studies instead, should consider the use of more improved camera systems with features that can extract both RGB and HSI simultaneously (Mesa and Chiang, 2021). Another study by Ucat and Cruz explored the use of image processing with a deep learning to grade banana according to their specifications (Ucat and Dela Cruz, 2019). The trained, validated, and test data by CNN model was more than 90% in all four classes of bananas (). The suggested CNN grading system in the tensor flow model can be commercially developed (Ucat and Dela Cruz, 2019).

Portable spectrometers and real-time online detection devices have recently developed for fruits and vegetables quality assessment. Portable devices are handheld, light weight, compact size and they are applied for in-field measurements (Sohaib et al., 2020). The combination of portable NIR device with MSC-PCA+LDA model was used to evaluate pineapple quality. These models were recommended to be developed in mobile phone while PLS regression model provided 85% accuracy (Amuah et al., 2019). Subedi et al. (Subedi and Walsh, 2020) evaluated three hand held portable near infrared spectroscopy (F750, Micro NIR and Scio v1.2) in the detection of dry matter content (DMC) in avocado fruit. The second derivative spectra were recorded for the intact and skin removed avocado fruit for reflectance and interactance optical geometry. The best results of prediction obtained from the F750 instrument using the interactance mode at 720-975 nm with R^2^p of 0.71 and 0.88 for intact and skin removed fruits respectively (Subedi and Walsh, 2020). Real time monitoring device was designed as sensor which can function in all post-harvesting states to control the shelf life of fruits and vegetables such as lettuce. The device found to be the feasible for controlling the behavior of the crop during the post handling chain (Torres-Sánchez et al., 2020). Fruits and vegetables including banana, orange and apple were well sorted according to their external appearance by using real time online system with artificial intelligence (Tata et al., 2022). For quality categorization, machine learning models such as CNN and image processing were performed. This real time system was created in android and can be deployed in market robots where checking of huge number of products is required (Tata et al., 2022).

## 5 Conclusion and future prospects

Non-destructive spectral measurement has emerged as a prominent solution in the agricultural sector. With the introduction of spectral measurements, there has been rapid progress in analyzing both the internal and external characteristics of tropical fruits and vegetables in a low-cost, accurate, real-time, and fast manner (Ali et al., 2017). Techniques based on FTIR, NIR, and Raman spectroscopy require simple steps to prepare samples prior to analysis (Abbas et al., 2020). In contrast to other imaging techniques such as computer vision, acoustic approaches, electric noses, and fluorescence, HSI uses spectral and spatial data to assess different parameters concurrently (Lu et al., 2020). The spectral measurements presented in this review have shown potential applications for a diverse range of tropical fruits and vegetables for the monitoring and detection of quality attributes such as SSC, TSS, TA, color, size, defects, and texture, which is particularly important for fruit and vegetable processors, food safety agencies, and consumer demands.

Significant advancements in non-destructive spectral measurement technology have occurred recently, including the development of portable spectrometers for real-time and field applications. The combination of spectral measurements and chemometric techniques is a powerful tool for multivariate data analysis, mainly in the improvement of models needed for classification and estimation of quality. A practical case study of Metlenkin et al. (Metlenkin et al., 2022) in the identification and classification of Hass avocado defects before and after storage by HSI and chemometrics. The PLSDA and SIMCA were selected as chemometric methods for multivariate data discrimination and classification. To increase the final model accuracy the calibration was performed by selecting the region of interest. The results revealed the high potential of SIMCA during both modelling and test validation with 100% accuracy. Furthermore, the integration of spectral measurements with deep learning and machine learning technology is rapidly expanding in order to improve quality control accuracy while overcoming the challenges associated with chemometrics such as spectral variability, spectrometer heterogeneity, changing environmental conditions, and infrared spectral data with high noise. The revolution in agriculture and the adaptation of numerous tropical plants to regions outside of their natural range have muddied their classification, and little is known about what properly defines and distinguishes tropical fruits and vegetables from their temperate counterparts. Therefore, there is confusion associated with those studies that reported the classification of tropical fruits and vegetables as an important factor to consider when examining the distinctive quality indicators of these crops. Taking into accounts all of the merits and demerits of non-destructive spectral measurements for the quality monitoring of tropical fruits and vegetables, the use of an adequate number of samples, different cultivars of the fruit and increasing the quality attributes to predict can help to develop robust models that emphasize the variability of tropical fruits and vegetables in terms of size and shape, skin thickness, and growing conditions.

## Author contributions

Conceptualization: UA, B-KC. Methodology: UA, TB, MF, MK and IB. Investigation: UA, TB and B-KC. Writing and reviewing: UA, TB, MF and B-KC. Supervision: B-KC. All authors contributed to the article and approved the submitted version.
